# *Phasmarhabditis zhejiangensis* sp. nov. (Nematoda: Rhabditidae), a new rhabditid nematode from Zhejiang, China

**DOI:** 10.1371/journal.pone.0241413

**Published:** 2020-11-09

**Authors:** Chao-nan Zhang, Qi-zhi Liu

**Affiliations:** College of Plant Protection, China Agricultural University, Beijing, China; INSERM U869, FRANCE

## Abstract

A new nematode species of the genus *Phasmarhabditis* was isolated from the body surface of a slug (*Philomycus bilineatus* Benson, PB). Morphological and molecular analyses confirmed this nematode as a new species. The nematode was named *Phasmarhabditis zhejiangensis* sp. nov. (Nematoda: Rhabditidae) and is dioecious. In males, the open bursa with genital papillae is characterized by the formula 1-1-1-2-1-3, and the spicule length is 58μm. In female, the vulva is located approximately in the middle of the body. The nematode belongs to papillosa group because of its tail shape pointed with filiform tip. The phasmids are rod-shaped. The posterior anus is slightly swollen. *P*. *zhejiangensis* was further characterized by internal transcribed spacer (ITS), 18S rDNA and 28S rDNA sequences. After the sequencing results were compared with sequences available from the National Center for Biotechnology Information (NCBI), the maximum similarities of ITS, 18S and 28S sequences were 89.81%, 96.22% and 95.28%, respectively. Phylogenetic analyses placed *Phasmarhabditis zhejiangensis* sp. nov. in the genus *Phasmarhabditis*.

## Introduction

Slugs are widespread and increasingly harmful to crops [1]. Studies have shown that slugs can cause significant damage to winter wheat in the UK [2, 3]. Similarly, slugs damaged many horticultural crops and cause yield and quality losses [4, 5]. Surprisingly, only one slug per square meter can cause severe damage to rapeseed (Brassicaceae) seedlings [6]. *Philomycus bilineatus* (Benson) has caused serious harm to a variety of vegetables and fruit trees in Ningbo, Zhejiang province, China, such as cabbage, cauliflower, spinach, rape, lettuce, potato, asparagus, mulberry, peach trees, pear trees and so on [7–10].

Biological control plays an increasingly important role in agricultural systems. Entomopathogenic nematodes (EPNs) have become a widely used biological resource for pest control [11]. Some nematodes in the families of Rhabditidae, Agfidae, Alaninematidae, Alloionematidae, Angiostomatidae, Cosmocercidae, Diplogasteridae and Mermithidae are related to slug and other mollusk control [1].

The genus *Phasmarhabditis* in the Rhabditidae family has been reported to be successfully used for slug control [1, 4]. To date, thirteen nematode species in the *Phasmarhabditis* genus had been recorded, including *P*. *hermaphrodita*, *P*. *neopapillosa*, *P*. *tawfiki*, *P*. *bonaquaense*, *P*. *californica*, *P*. *huizhouensis*, *P*. *apuliae*, *P*. *bohemica*, *P*. *meridionalis*, *P*. *safricana*, *P*. *papillosa*, *P*. *circassica* and *P*. *clausliiae* [12–25].

Only *P*. *hermaphrodita* (Rhabditida: Rhabditidae) has been marketed under trade name Nemaslug® (MicroBio Ltd, UK) even though 13 species of *Phasmarhabditis* have been reported [1, 4, 26–30]. Therefore, discovering and developing new nematode species associated with slug and other mollusk control are crucial.

Fortunately, a species of nematode isolated from the body surfaces of slugs (*P*. *bilineatus* Benson, PB) was identified in this study as a new species in the genus *Phasmarhabditis* (Rhabditidae) by the methods of morphological identification and molecular biological analysis.

## Material and methods

### Collection and culture of nematodes

Slugs (*P*. *bilineatus* Benson, PB) were collected from vegetable gardens containing cruciferous cabbage and canola in the Yinzhou district of Ningbo city, Zhejiang province, China, lies between 121°08' -121°54' and 29°37'-29°57', where belongs to subtropical monsoon climate. The annual average temperature is 16.4°C, and the annual average air relative humidity is about 66% RH. The slug survival environment is mild and humid. The survival temperature and relative humidity for the slug are 15–25°C and 60–75% RH, respectively. The climatic conditions in this area are suitable for the slug living.

The gardens where the slugs were collected from belong to farmers who welcome people into their gardens to collect slugs, which successfully decreases the slug populations. Therefore, no special permission was needed except for a verbally informal notification regarding slug collection in their gardens.

The original nematodes were observed on the body surfaces of the slugs in different age of juveniles and adults. Then, a series of work such as collection (by rinsing the surface of the slugs with tip water), isolation, propagation (in NGM), purification and identification on the nematodes were carried out successively.

The NGM medium (nematode growth medium with NG agar and M9 buffer) was prepared according to Brenner et al. [18]. The nematodes that propagated in the medium were washed off and collected into plastic bags in density of 3000 IJs/ml. For keeping nematode longer period of life, superabsorbent polymer (1.5 g/100 ml) was added into nematode suspension in the plastic bags and put them in a refrigerator at 4°C.

### Light and scanning electron microscopy

Fresh heat-killed nematode adults fixed in triethanolamine formalin (TAF) and subsequently processed with glycerin by Seinhorst's method [31] were used for microscopy observation. Then, the nematodes were observed by a stereoscope (Leica S8AP0) and a light microscope (Leica DM2500), photos were collected, and the data were measured with a custom hardware configuration.

The scanning electron microscope (SEM) used in this study was a HITACHI S-3400N located at China Agricultural University. The adult nematodes used for scanning electron microscopy were rinsed with phosphate-buffered saline (PBS) and fixed at 4°C for 24 hours with 3% glutaraldehyde solution. They were then post fixed with 2% osmium tetroxide solution for 12 hours at 25°C, dehydrated in a graded ethanol series, critical point dried with liquid CO_2_, mounted on SEM stubs, and coated with gold [32].

According to observations of nematodes under a light microscope and an SEM, we made a hand-drawn diagram of nematode morphology.

### Molecular analysis

According to the traditional method [33], DNA was extracted from a single nematode. Three repetitions of DNA extraction were carried out with 3 cultural batches. In each repetition, 10 adult individuals were picked out as 10 replications. Totally, thirty adult nematodes’ DNA in different cultural batches were extracted. Each nematode was placed into an Eppendorf (EP) tube containing 20 μl extraction buffer, including 17.7 μl of PCR-grade water, 2 μl of 10×PCR buffer with MgCl_2_, 0.2 μl of 1% Tween-20, and 0.1 μl of protease K. After the above steps were completed, the EP tube was placed into a freezer at -80°C and frozen for 10 minutes. Then, the EP tube was quickly put into a water bath at 65°C. After 90 minutes of protease K digestion, the temperature was raised to 95°C through the water bath. After 10 minutes in the water bath, the protease K was inactivated.

Then, the EP tube was quickly placed on ice to cool and centrifuged at 12000 r/min for 2 minutes, after which 1 μl of supernatant was used for PCR. The PCR consisted of 25 μl master mix including 12.5 μl of Taq mix, 1μl of forward primer and and 1 μl of reverse primer, in addition to 1 μl of the DNA template. Finally, add 9.5 μl sterile water.

The sequencing results were analyzed by us using the NCBI database, DNAMAN and MEGA-X.

### Nomenclatural acts

The electronic edition of this article conforms to the requirements of the amended International Code of Zoological Nomenclature, and hence the new names contained herein are available under that Code from the electronic edition of this article. This published work and the nomenclatural acts it contains have been registered in ZooBank, the online registration system for the ICZN. The ZooBank LSIDs (Life Science Identifiers) can be resolved and the associated information viewed through any standard web browser by appending the LSID to the prefix "http://zoobank.org/". The LSID for this publication is: urn: lsid: zoobank. org: pub: F2B458AC-D13A-4F11-9222-C6C9892E2459. The electronic edition of this work was published in a journal with an ISSN, and has been archived and is available from the following digital repositories: PubMed Central, LOCKSS.

### Phylogenetic trees

The sequences were aligned in MEGA-X using ClustalW algorithm with default parameters. The phylogenetic trees were constructed also in MEGA-X using the neighbor-joining (NJ) method. Branch support was estimated by bootstrap analysis with 500 replicates.

## Results

**Fig 1:**
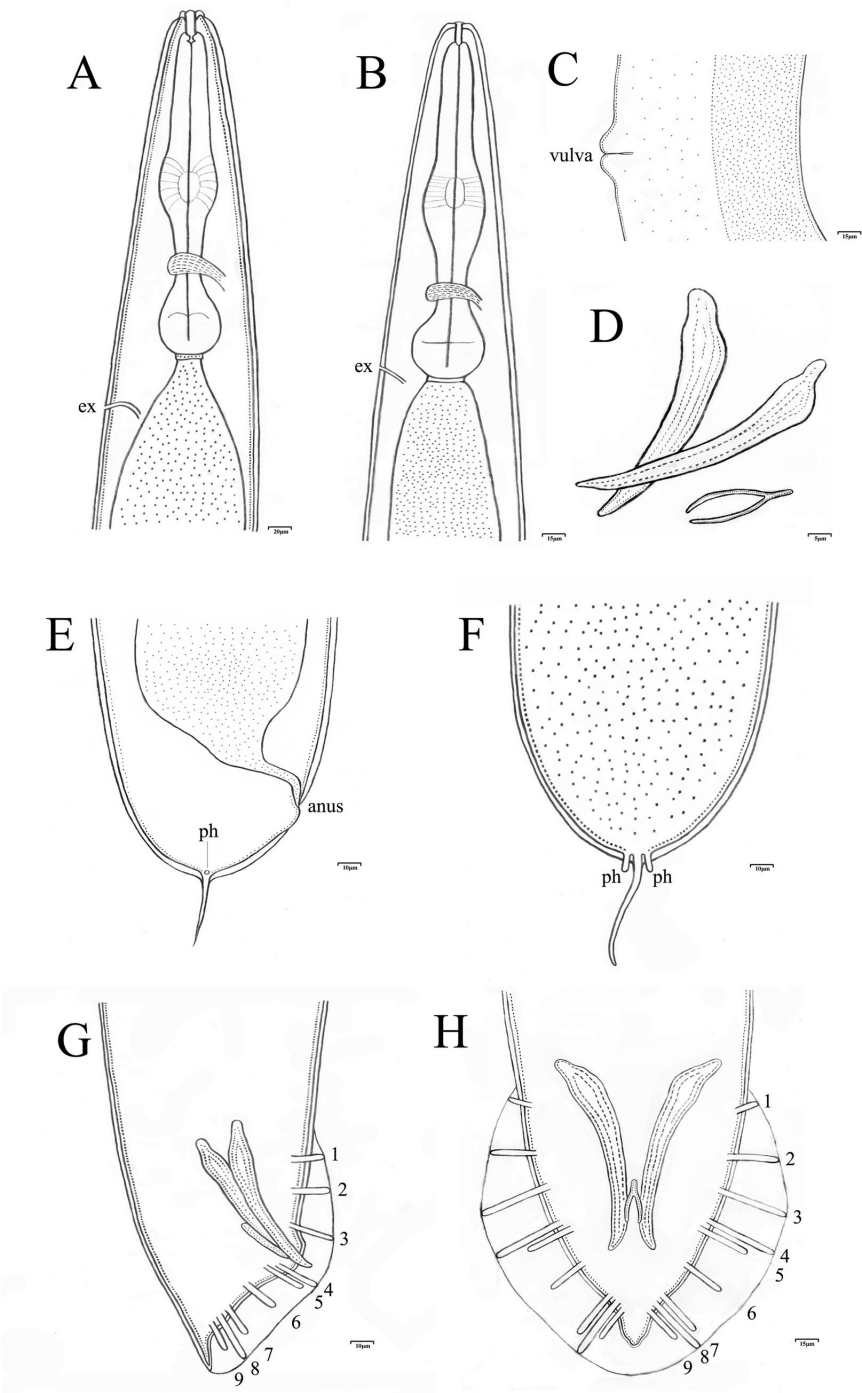
Hand drawings of *Phasmarhabditis zhejiangensis* sp. nov. (A) Head of female, (B) Head of male, (C) Vulva of female, (D) Spicule and gubernaculum, (E) Lateral view of female tail, (F) Ventral view of female tail, (G) Lateral view of male tail, (H) Ventral view of male tail.

**Fig 2:**
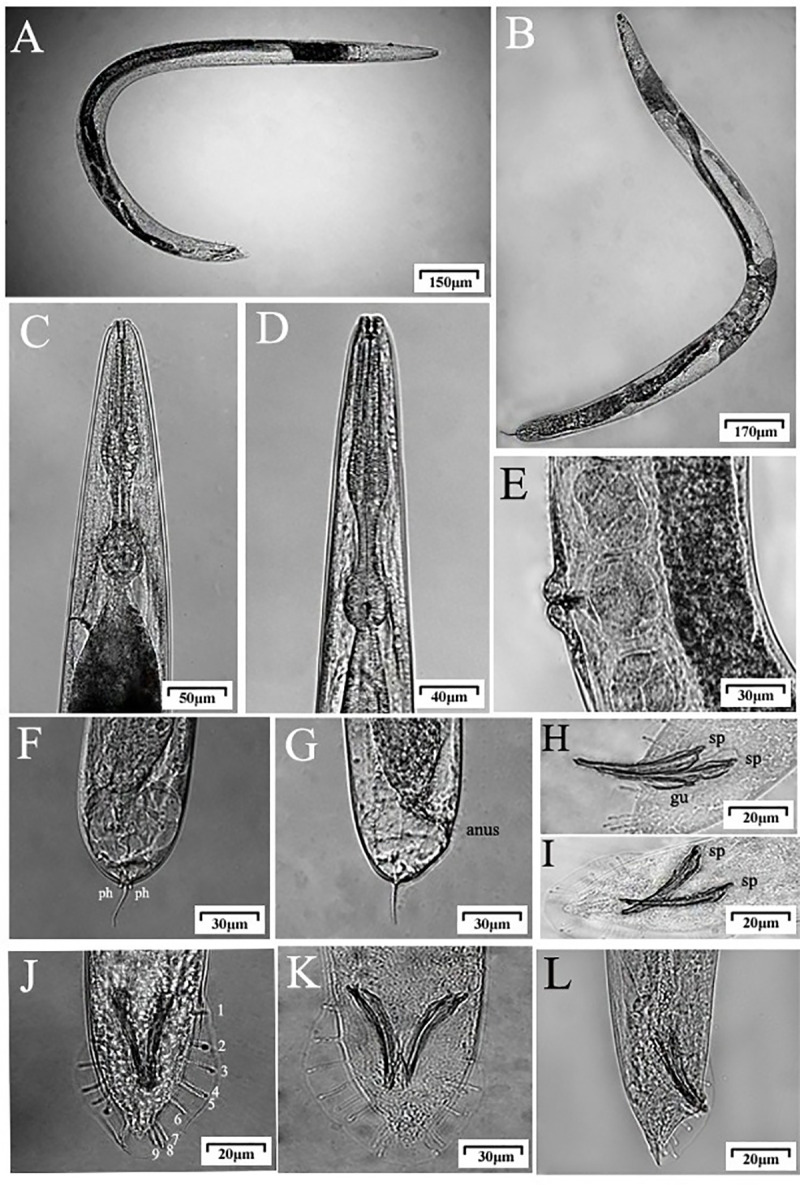
Optical micrographs of *Phasmarhabditis zhejiangensis* sp. nov. (A) Male, (B) Female, (C) Head of female, (D) Head of male, (E) Vulva, (F) Ventral view of female tail, (G) Lateral view of female tail, (H) Spicule and gubernaculum, (I) Spicule, (J and K) Ventral view of male tail, (L) Lateral view of male tail.

**Fig 3:**
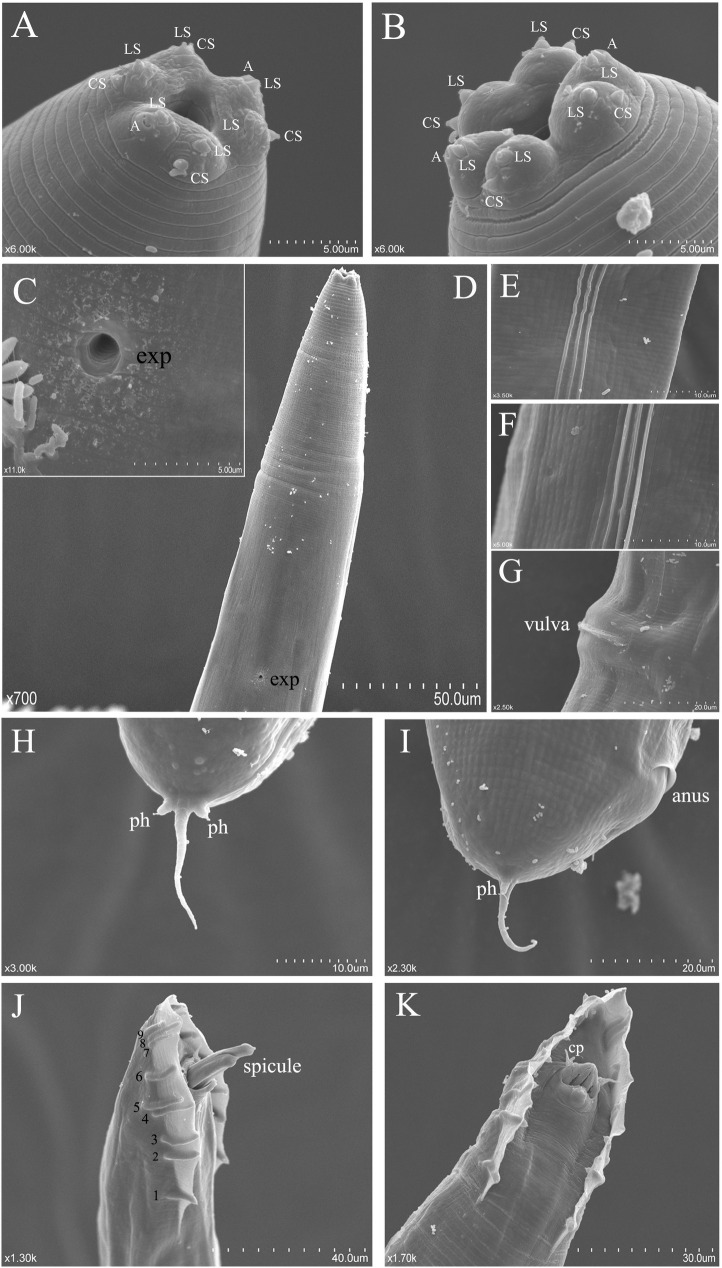
Scanning electron micrograph of *Phasmarhabditis zhejiangensis* sp. nov. (A) Head of male (LS: labial sensilla; CS: cephalic sensilla; A: amphid), (B) Head of female, (C) Enlarged view of excretory pore, (D) Excretory pore in the anterior region of the body in both the male and female, (E) Lateral field of female, (F) Lateral field of male, (G) Lateral view of the vulva of the female, (H) Ventral view of the female tail (ph: phasmid), (I) Lateral view of the female tail, (J) Lateral view of the male tail, (K) Ventral view of the male tail (cp: cloacal papillae).

The new species nematode described in this paper is named ***Phasmarhabditis zhejiangensis* sp. nov.** urn: lsid: zoobank.org: act: F8111D77-AF37-4518-BB6E-FC9818CF1708. The main morphological characteristics of this nematode are shown in Figs 1–3.

### Measurements

The eighteen measured values of the main morphological characteristics of *P*. *zhejiangensi*s, including body length, greatest body diameter, esophageal length and so on, are shown in Table 1.

**Table 1:** Morphological data of *Phasmarhabditis zhejiangensis* sp. nov.

Character	Males		Females	Dauer juveniles
	Holotype	Paratypes	Paratypes	
**n**	-	20	20	20
**L**	1667	1486.4±241.7 (1014.6–1842.7)	1911.4±294.5 (1440.7–2354.9)	610.6±84.1 (513.7–663.0)
**a**	20.3	20.3±2.0 (17.0–25.6)	16.6±1.2 (14.1–18.8)	17.1±1.51 (16.1–18.9)
**b**	8.5	7.5±0.9 (5.6–8.8)	7.7±0.9 (6.3–10.1)	—
**c**	35.5	31.0±7.0 (20.0–48.7)	26.0±4.7 (19.9–33.5)	7.7±0.9 (5.6–9.4)
**c’**	1.7	1.1±0.2 (0.8–1.4)	1.3±0.3 (0.9–1.9)	2.3±0.2 (1.8–3.5)
**V**	—	—	53.9±1.4 (50.8–57.2)	—
**Greatest body diameter**	82	74.3±15.0 (50.9–94.1)	115.1±19.5 (79.1–154.7)	35.8±4.8 (31.6–41.1)
**Esophageal length**	195	197.3±12.3 (176.3–223.8)	247.7±21.5 (214.2–285.5)	—
**Distance from anterior end of body to nerve ring**	200	161.7±14.1 (133.9–179.1)	195.0±18.7 (166.9–228.5)	—
**Distance from anterior end of body to excretory pore**	218	208.8±19.2 (179.4–237.6)	243.7±22.9 (201.7–289.5)	—
**Distance from anterior end of body to vulva**	—	—	1031.1±152.8 (783.7–1264.2)	—
**Distance from vulva to anus**	—	—	809.9±145.3 (549.3–1013.2)	—
**Anal body diameter**	40	43.5±8.3 (30.7–62.0)	56.1±10.2 (35.8–75.3)	—
**Tail length**	47	49.9±12.7 (31.2–88.6)	73.5±6.3 (59.9–83.5)	79.4±10.9 (69.4–92.2)
**Length of female pointed tip**	—	—	35.6±5.0 (24.8–47.4)	—
**Spicule length**	61	58.0±5.1 (48.3–68.1)	—	—
**Gubernaculum length**	23	20.1±2.6 (15.7–28.0)	—	—
**Gubernaculum length (% of spicule length)**	37.7%	34.8±4.0 (25.6–42.8)	—	—

All measurements are presented in μm and in the form mean ± s. d. (range) (n = 20).

### Description

#### Female

The female head has six labial sensilla (a total of three pairs) and four cephalic sensilla, but compared with male, the female has a more noticeable head and more obvious labial and cephalic sensilla. Each of the two lateral lips without cephalic sensilla has a prominent amphids opening (Fig 3B). The body of the female is more robust than that of the male. The esophageal length is 247.7 μm. Nerve ring encircling posterior half of isthmus. The excretory pore and the lower end of the esophageal bulb are basically in a horizontal position. The distance from anterior end of body to nerve ring and excretory pore is 195 μm and 243.7 μm, respectively. The former is about 80% of the latter (Table 1). The vulva with a transverse slit is located approximately in the middle of the body, and the vulval lips are protruding and amphidelphic (Figs 1C, 2E and 3G). Pairs of ovaries are symmetrical around the vulva. The female thins from the vulva towards both ends. The tail shape is characteristic of the papillosa group, which is pointed with filiform tip, and two short, rod-shaped, conspicuous phasmids appearing in pairs (Figs 1F, 2F and 3H). The posterior anus is slightly swollen and slightly wider and longer than the anal body width (Figs 1E, 2G and 3I).

#### Male

The male head is flat. There are four cephalic sensilla and six labial sensilla, each two forming a pair for a total of three pairs. The sensilla of the head and lips are not prominent. Similar to the female, the male also have distinct amphids opening on two lateral lips of the head region (Fig 3A). The esophageal length is 197.3 μm. Nerve ring encircling posterior half of isthmus. The excretory pore is located just behind the esophageal bulb, the distance from anterior end of body to nerve ring is about 77% of the distance from anterior end of body to the excretory pore (Table 1). The male tail has and open bursa with nine pairs of genital papillae (1+1+1+2+1+3) (Figs 1G–1H and 3J). The spicules are 58 (48–68) μm long in average (Figs 1D and 3J, Table 1), and the gubernaculum is “Y” shaped (Figs 1D, 1G, 1H and 2H). The anterior end of the spicules is slightly enlarged and then tapered towards the end. There are two long and tapering cloacal papillae which are located in ventral side, near the opening of spicules, symmetrically distributed in pairs behind the abdomen and adjacent to the spicules (Fig 3K). The tail is short and conical, extending to a pointed end and bending towards the ventral side.

#### Dauer juvenile

Heat-killed nematodes are linear. The body of dauer juveniles is short, with the body tapering from the middle to both ends, the body length of the female is 513.7–663.0 μm, the greatest body diameter is 31.6–41.1 μm. The sheaths of most sheathed juvenile form semicircular folds on the surface and are not significantly separated from the worm. The lateral fields begin at the posterior of the head and ends at the tail region. There are two prominent wide ridges with three incisures. The internal structure of the body is clearer than that of the second instar larva. The lip region is relatively flat. The tail is long and tapered, and the tail length is 69.4–92.2 μm.

#### Type material

Holotype male, paratype males and paratype females will be sent to USDA Nematode collection and the Canadian National Collection of Nematodes for permanent preservation.

#### Diagnosis and relationships

*Phasmarhabditis zhejiangensis* sp. nov has the following remarkable characteristics. During the culture of *P*. *zhejiangensis* in the NGM medium, the male is distinct. The body contours of female and male are very large. The body length of the female is 1440.7–2354.9 μm, the “a” value is 14.1–18.8, and the greatest body diameter is 79.1–154.7 μm. The body length of the male is 1014.6–1842.7 μm, the “a” value is 17.0–25.6, and the greatest body diameter is 50.9–94.1 μm. The female tail suddenly becomes sharp and thin, forming a pointed tail with filiform tip, and the length of the tail is 59.9–83.5μm. The phasmids are very obvious, appearing in pairs on both sides of the tail. The male tail also has an open bursa with nine pairs of genital papillae (1-1-1-2-1-3). Specially, a pair of symmetrically distributed cloacal papillae are found near the opening of the spicules in the ventral side of the male, adjacent to the spicules.

*P*. *zhejiangensis* is similar to *P*. *safricana*, *P*. *bonaquaense*, *P*. *meridionalis*, *P*. *papillosa* and *P*. *huizhouensis* in terms of female’s tail pointed with a filamentous tip terminus and males’ genital papillae with 1-1-1-2-1-3 characteristics. Based on the tail shape, *P*. *zhejiangensis* and above mentioned species belong to the papillosa group and follows by comparing only the species from this group.

The female tail of *P*. *zhejiangensis* is similar to that of *P*. *safricana* [23], but the tail phasmids of *P*. *safricana* is not obvious. Besides, the body size of *P*. *zhejiangensis* is larger than that of *P*. *safricana* (Female: length: 1911 vs 1598 μm, greatest body width: 115 vs 104 μm; Male: body length: 1486 vs 1477 μm, greatest body width: 74 vs 65 μm), and the tail length of *P*. *zhejiangensis* is significantly longer than that of *P*. *safricana* (Female:73 vs 61 μm; Male: 50 vs 39 μm). The lengths of the spicules and gubernaculum of *P*. *zhejiangensis* are smaller than those of *P*. *safricana* (spicule: 58 vs 61 μm; gubernaculum: 20 vs 32 μm).

The body size of *P*. *zhejiangensis* is significantly smaller than that of *P*. *bonaquaense* [16] (Female: length: 1911 vs 2349 μm, greatest body width: 115 vs 136 μm; Male: body length: 1486 vs 1829 μm, greatest body width: 74 vs 90 μm). The spicule and gubernaculum on male of *P*. *zhejiangensis* are observably smaller than those of *P*. *bonaquaense* at 58 vs 77 μm and 20 vs 36 μm, respectively.

The body type of *P*. *zhejiangensis* is slightly larger than that of *P*. *meridionalis* [22] (Female: length: 1911 vs 1612 μm, greatest body width: 115 vs 94 μm; Male: body length: 1486 vs 1317 μm, greatest body width: 74 vs 60 μm), and the tail length is also longer than that of *P*. *meridionalis* (Female: 73 vs 68 μm; Male: 50 vs 32 μm). However, the “V” value is slightly smaller than that of *P*. *meridionalis* at 53.9 vs 56.1 and the spicule and gubernaculum are significantly shorter than those of *P*. *meridionalis* (spicule: 58 vs 76 μm; gubernaculum: 20 vs 43 μm).

Similarly, the body size of *P*. *zhejiangensis* is slightly larger than that of *P*. *papillosa* [17, 24] (Female: length: 1911 vs 1590 μm, greatest body width: 115 vs 81 μm; Male: body length: 1486 vs 1233 μm, greatest body width: 74 vs 61 μm), and no significant difference in the lengths of the spicules and gubernaculum between the two species, but the tail length of the females of *P*. *zhejiangensis* is significantly shorter than that of *P*. *papillosa* (73 vs 106 μm). In addition, the V value of *P*. *zhejiangensis* is larger than that of *P*. *papillosa* (53.9 vs 49.9).

The morphological characteristics of *P*. *zhejiangensis* are most similar to those of *P*. *huizhouensis* [19]. Comparing the two species, the body length between *P*. *zhejiangensis* and *P*. *huizhouensis* is no significant difference, but there has difference in the body width (Female: length: 1440.7–2354.9 vs 1333.1–2341.2 μm, greatest body width: 50.9–94.1 vs 85.5–171.0 μm; Male: body length: 1014.6–1842.7 vs 907.9–1669.2 μm, greatest body width: 79.1–154.7 vs 65.1–114.0 μm). In addition, the lengths of the spicule and gubernaculum of *P*. *zhejiangensis* are significantly smaller than those of *P*. *huizhouensis*. The length of the spicule is 48.3–68.1 μm and the length of the gubernaculum is 15.7–28.0 μm with *P*. *zhejiangensis*, while in *P*. *huizhouensis*, they are 61.07–81.88 μm and 29.9–41 μm, respectively. In *P*. *zhejiangensis*, the percentage of the length of the gubernaculum to the spicule is 34.8%, while that of *P*. *huizhouensis* is 50.4%. The difference is obvious and easy to distinguish.

**Fig 4:**
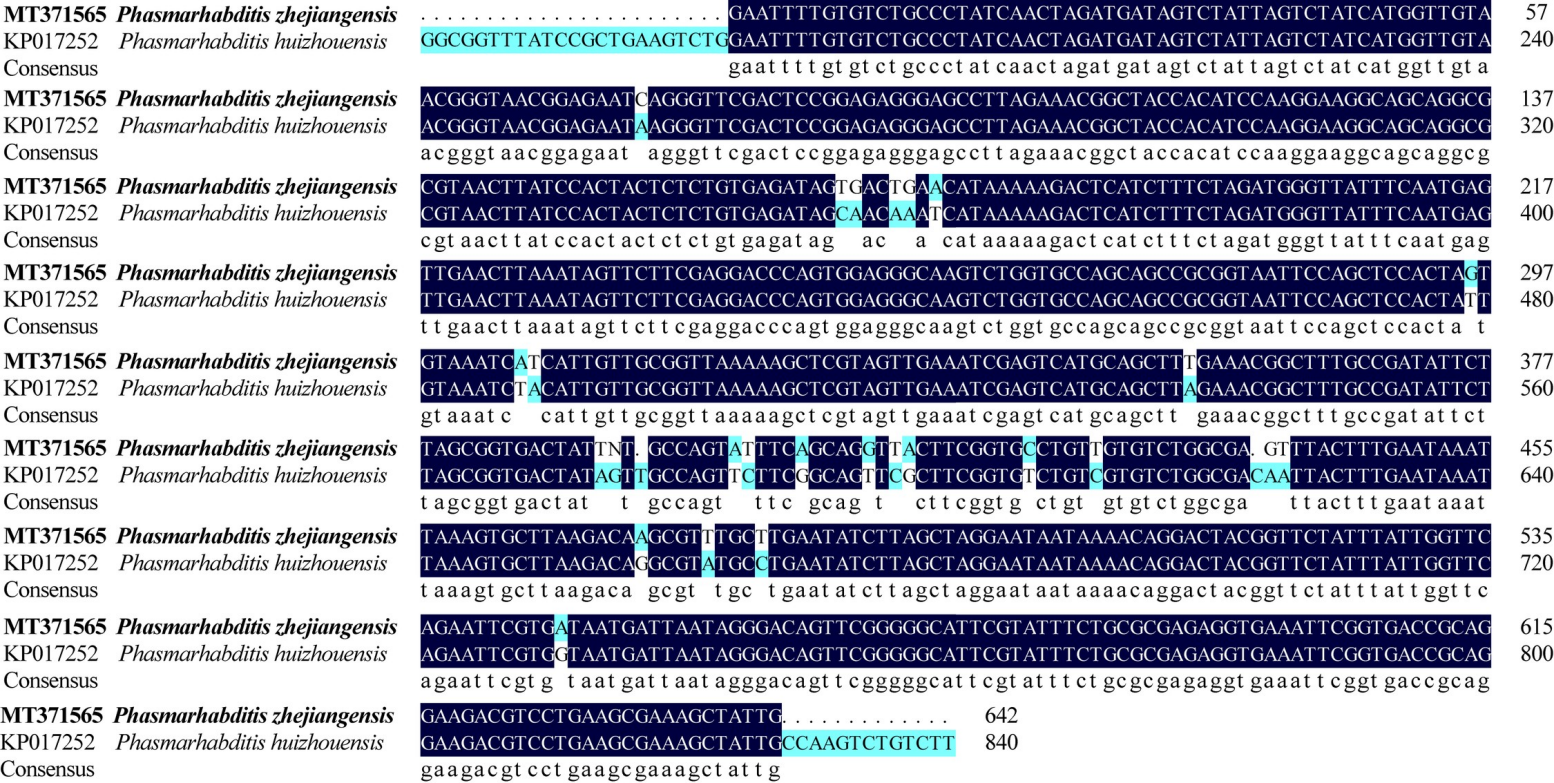
Comparison of 18S sequence between *Phasmarhabditis zhejiangensis* sp. nov and *Phasmarhabditis huizhouensis*.

**Fig 5:**
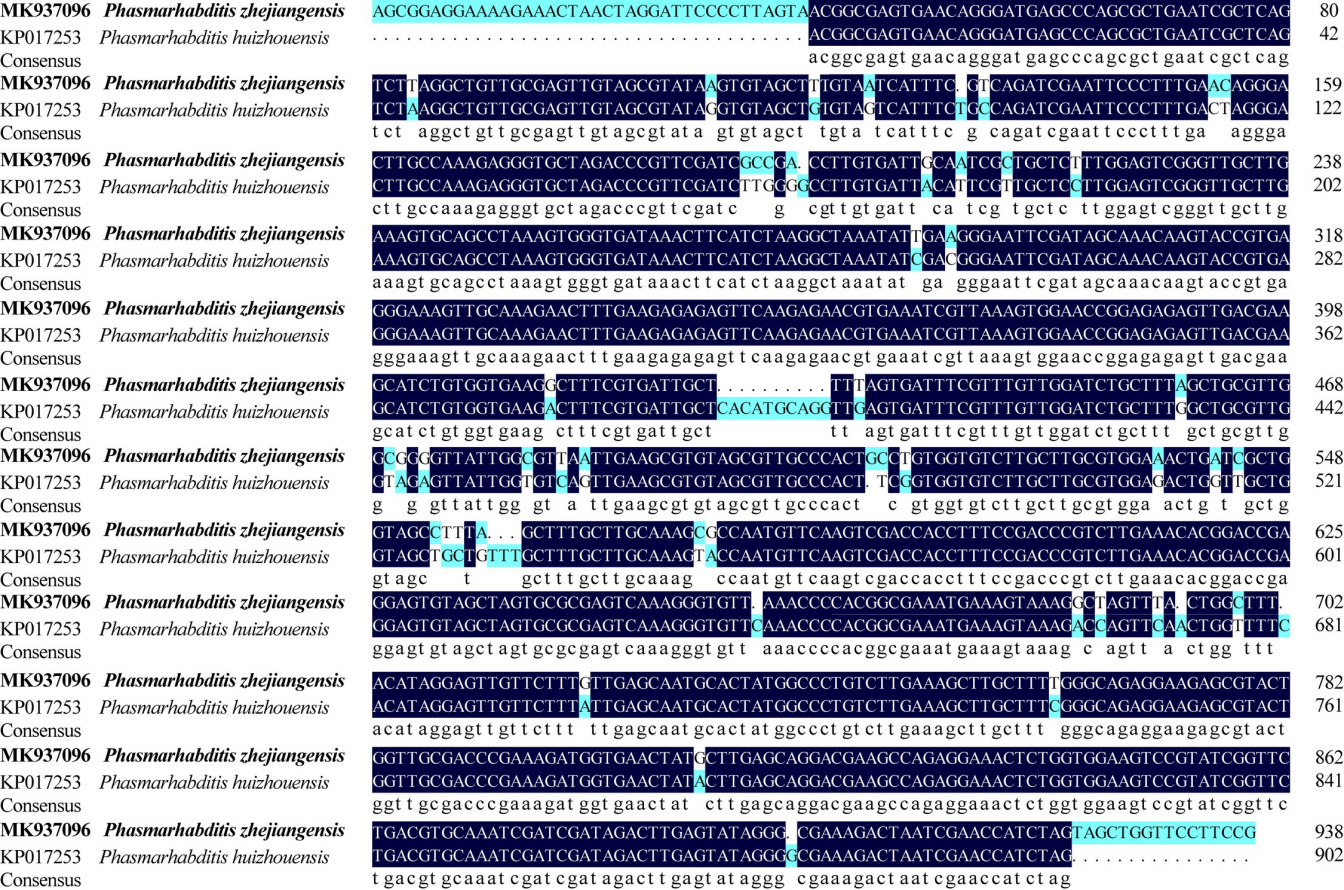
Comparison of 28S sequence between *Phasmarhabditis zhejiangensis* sp. nov and *Phasmarhabditis huizhouensis*.

*P*. *zhejiangensis* and *P*. *huizhouensis* are not similar in molecular analysis as well. DNAMAN was used to compare the 18S and 28S sequences of these two nematodes. It was found that the sequence similarity of these two nematodes in 18S (Fig 4) and 28S (Fig 5) was 85.24% and 87.58% respectively, which proving the two nematodes in different species.

#### Molecular identification

The ITS, 18S, and 28S sequencing results for *P*. *zhejiangensis* were blasted against the NCBI database, and the highest identities were 89.81%, 96.22%, and 95.28%, respectively. The three sequences were deposited in GenBank with accession numbers MK542667, MT371565 and MK937096, respectively.

**Fig 6:**
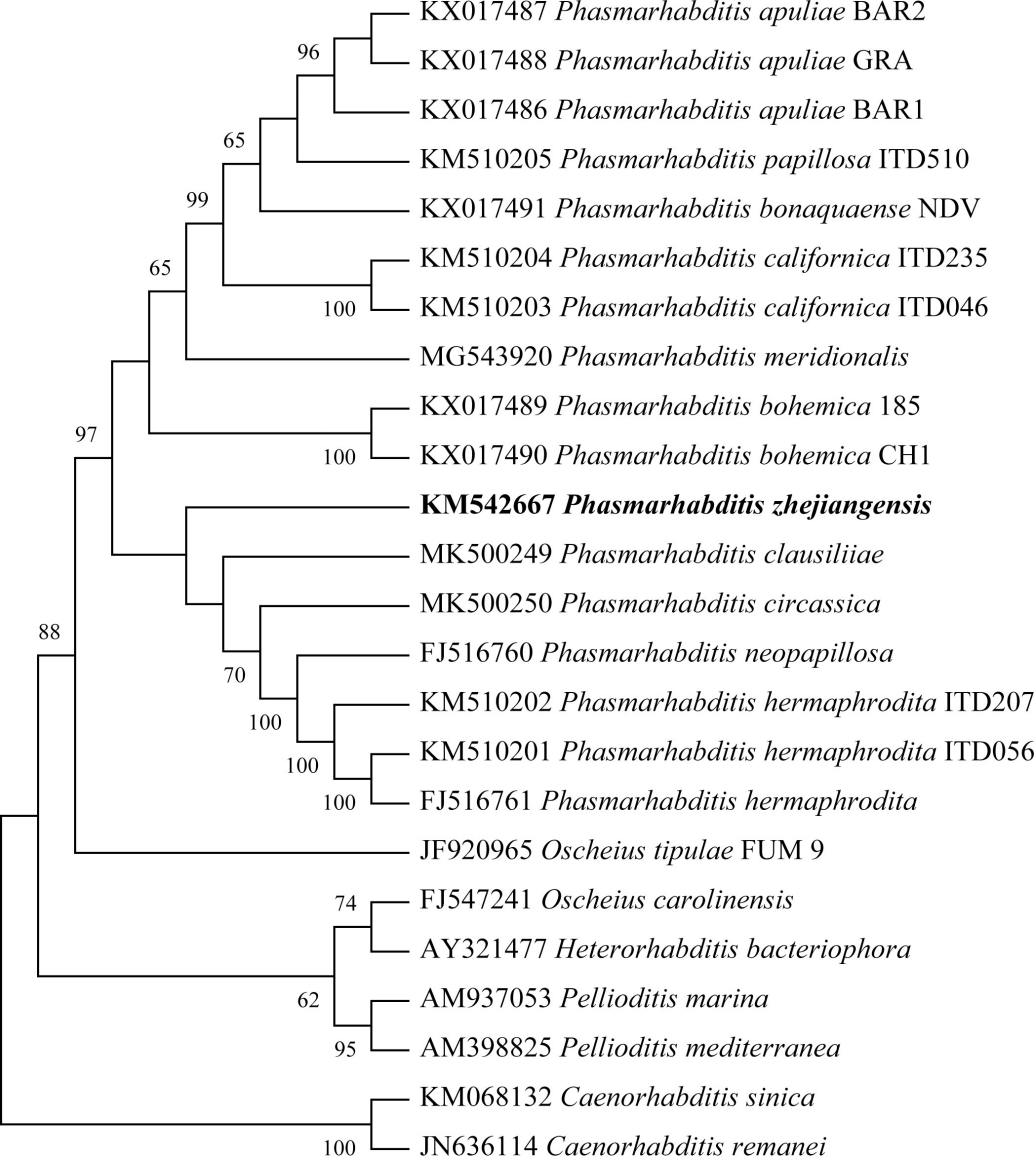
Molecular evolutionary tree based on the ITS sequence of *Phasmarhabditis zhejiangensis* sp. nov. and other *Phasmarhabditis* species (MEGA-X). Bootstrap support values are presented near nodes as NJ. Support values lower than 50% are not presented.

**Fig 7:**
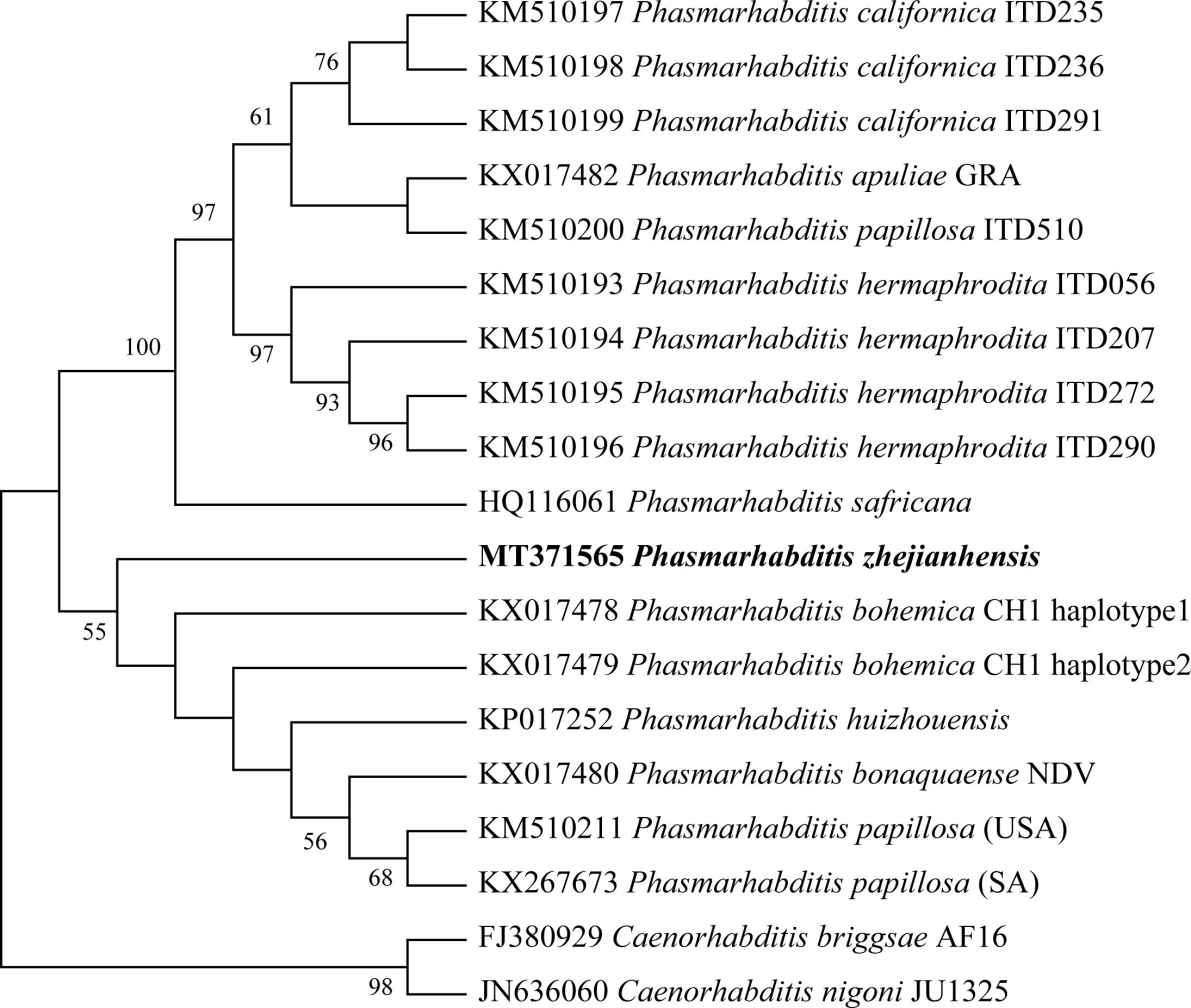
Molecular evolutionary tree based on the 18S rDNA sequence of *Phasmarhabditis zhejiangensis* sp. nov. and other *Phasmarhabditis* species (MEGA-X). Bootstrap support values are presented near nodes as NJ. Support values lower than 50% are not presented.

**Fig 8:**
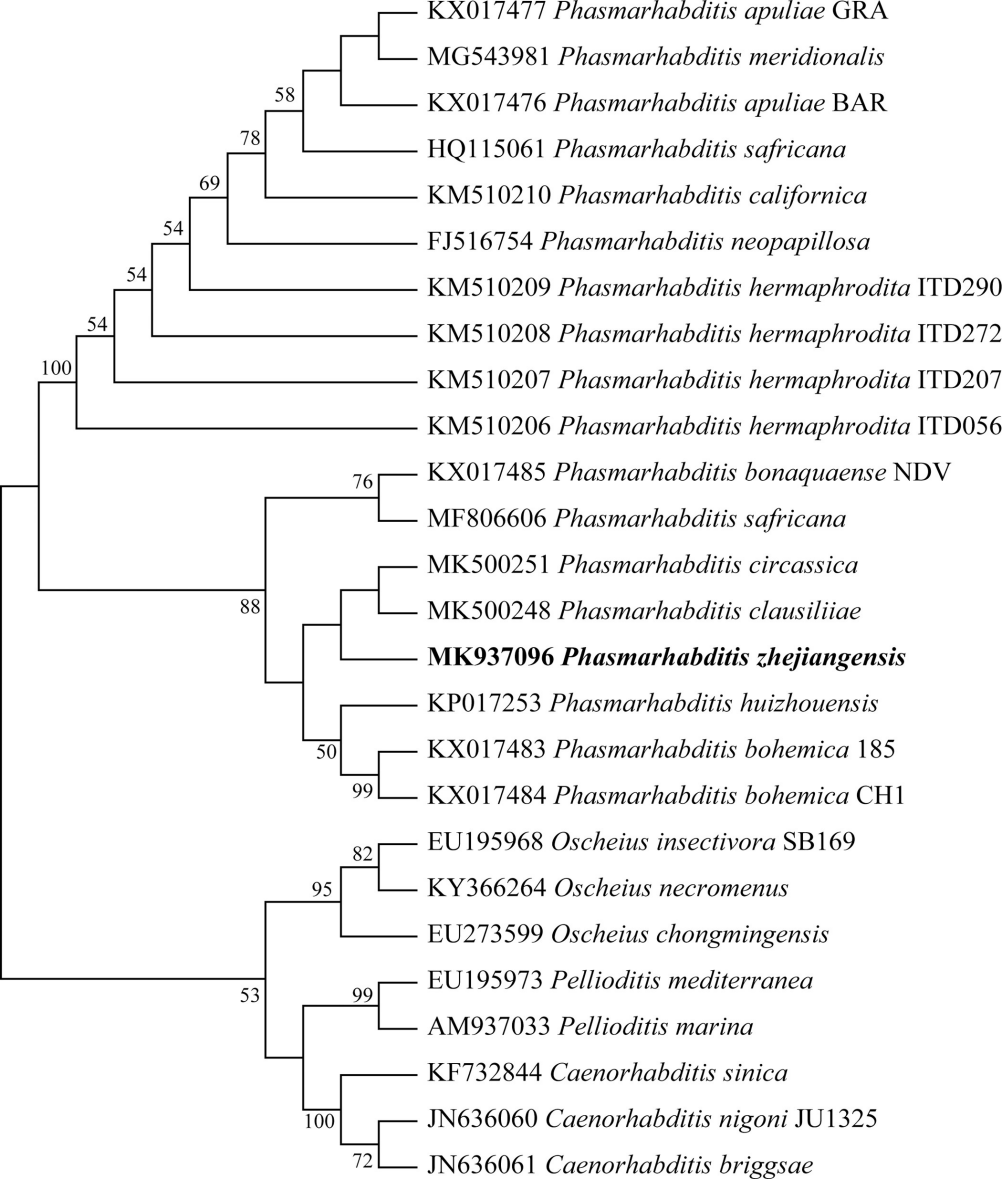
Molecular evolutionary tree based on the 28S rDNA sequence of *Phasmarhabditis zhejiangensis* sp. nov. and other *Phasmarhabditis* species (MEGA-X). Bootstrap support values are presented near nodes as NJ. Support values lower than 50% are not presented.

Phylogenetic trees of the ITS (Fig 6), 18S (Fig 7) and 28S (Fig 8) sequences were constructed by the neighbor-joining (NJ) method. The three molecular evolutionary trees showed a close evolutionary relationship between *P*. *zhejiangensis* and the nematode species in *Phasmarhabditis* genus with high bootstrap support. Thus, it can be generally confirmed that this nematode belongs to the genus of *Phasmarhabditis*, not to another genus and not the same species with neighboring species. Therefore, the studied nematode, *P*. *zhejiangensis* appears to be a new species of the genus *Phasmarhabditis*.

## Discussion

Accurate identification of nematode species is fundamental to using nematodes as a biocontrol factor and for further research in nematology. Morphological identification and molecular biological analysis are still very common and important methods for the classification of nematodes. In this paper, the main morphological characteristics of the tested nematodes were observed in depth, including the head, tail, vulva of the female, spicule, gubernaculum and genital papillae of the male, among others. Then, molecular biological analysis of ITS rDNA, 18S rDNA and 28S rDNA sequences was carried out, and the nematode was identified as a new species of the genus *Phasmarhabditis*.

Similar to EPN species in the families Steinernematidae and Heterorhabditidae, slug-parasitic nematode species in *Phasmarhabditis* are also important pathogenic agents for agricultural pest control [34]. In recent years, species of the genus *Phasmarhabditis* have been continuously reported and improved, which provides a basis for studying the relationships within this genus of nematodes as well as a theoretical basis for nematology and nematode taxonomy.

## Supporting information

S1 File (ZIP)
