# Supplementary material for: Phasmarhabditis zhejiangensis sp. nov. (Nematoda: Rhabditidae), a new rhabditid nematode from Zhejiang, China
Source: PLoS One. 2020 Nov 9;15(11):e0241413. doi: 10.1371/journal.pone.0241413 (PMC7652337; doi:10.1371/journal.pone.0241413)
Supplement: S1 File — (ZIP) [file pone.0241413.s001.zip › supporting information/Z3H93XY7_Highlights_edited_final.docx]

Highlights:

1. A new nematode species, *Phasmarhabditis zhejiangensis* sp. nov., was isolated from the body surface of a slug (*Philomycus bilineatus* Bonson, PB).

2. The morphological characteristics of *P. zhejiangensis* were observed under light and scanning electron microscopes, and hand drawings and morphological measurements were made.

3. Molecular analysis of *P. zhejiangensis* ITS, 18S and 28S sequences was performed to further support it as a new species.
