# Supplementary material for: Phasmarhabditis zhejiangensis sp. nov. (Nematoda: Rhabditidae), a new rhabditid nematode from Zhejiang, China
Source: PLoS One. 2020 Nov 9;15(11):e0241413. doi: 10.1371/journal.pone.0241413 (PMC7652337; doi:10.1371/journal.pone.0241413)
Supplement: S1 File — (ZIP) [file pone.0241413.s001.zip › supporting information/Z3H93XY7_Main_Document_edited_final.docx]

*Phasmarhabditis zhejiangensis* sp. nov. (Nematoda: Rhabditidae), a new rhabditid nematode from Zhejiang, China

**Chao-nan ZHANG, Qi-zhi LIU ^*^**

*Laboratory of Entomology and Nematology, College of Plant Protection, China Agricultural University, Beijing, 100193, China*

**Abstract**

A new nematode species of the genus *Phasmarhabditis* was isolated from the body surface of a slug (*Philomycus bilineatus* Bonson, PB). Morphological and molecular analyses confirmed this nematode as a new species. The nematode was named *Phasmarhabditis zhejiangensis sp. nov.* (Nematoda: Rhabditidae) and is dioecious. In males, the open bursa with genital papillae is characterized by the formula 1 1 1 2 1 3, and the spicule length is 58 μm. In females, the vulva is located approximately in the middle of the body, the end of the tail suddenly thins into a "small tail" with two short, rod-shaped phasmids, and the posterior anus is slightly swollen. *Phasmarhabditis zhejiangensis sp. nov.* was further characterized by internal transcribed spacer (ITS), 18S rDNA and 28S rDNA sequences. After the sequencing results were compared with sequences available from the National Center for Biotechnology Information (NCBI), the maximum similarity of ITS, 18S and 28S was 89.81%, 98.06% and 95.28%, respectively. Phylogenetic analyses placed *Phasmarhabditis zhejiangensis sp. nov.* in the genus *Phasmarhabditis.*

**Key words:** new species, description, optical and scanning electron microscopy, hand drawings, ITS, 18S, 28S

**Introduction**

Slugs are widespread and increasingly harmful to crops [[1](#文献1)]. Studies have shown that slugs can cause significant damage to winter wheat in the UK [[2](#文献2),[3](#文献3)]. Similarly, the damage caused by slugs to the yield and quality of many horticultural crops is worrisome [[4](#文献4),[5](#文献5)]. For example, shockingly, one slug per square meter can cause severe damage to rapeseed (Brassicaceae) seedlings [[6](#文献6)]. Potatoes are also severely damaged by slugs, with slugs capable of damaging every organ [[7](#文献7)-[9](#文献9)].

With biological control playing an increasingly important role in agricultural systems, nematodes, as a new biocontrol factor, have become a biological resource for pest control and are now widely used [[10](#文献10)]. Although several families of mollusk-parasitic nematodes have been reported, only *Phasmarhabditis hermaphrodita* (Rhabditida: Rhabditidae) has been marketed, under the trade name Nemaslug® (MicroBio Ltd, UK) [[1,](#文献1) [4](#文献4), [11](#文献11)-[15](#文献15)]. Therefore, the development and use of new species of nematodes associated with slugs and other mollusks are crucial. In recent years, nematodes of the genus *Phasmarhabditis* (Rhabditidae) have been reported. *P. phasmarhabditis* has been reported in North America, and *P. neopapillosa* was found to be most closely related to *P. hermaphrodita*. In addition, *P. papillosa*, *P. tawfiki*, *P. bonaquaense*, *P. californica* and *P. huizhouensis* have been reported successively [[16](#文献16)-[22](#文献22)]. Fortunately, we have also isolated a new species of nematode related to slugs through morphological identification and molecular biological analysis. This nematode was identified as a new species of the genus *Phasmarhabditis* (Rhabditidae).

**Material and methods**

**Collection and culture of nematodes**

This new species of nematode was isolated from the surface of a slug in Zhejiang Province, China. We washed the surface of the slug with water to collect nematodes, which then were purified in nematode growth medium (NGM, NG agar and M9 buffer) and exhibited considerable population growth.

The NGM medium was prepared according to Brenner *et al*. [[23](#文献23)] as follows: 3 g of NaCl, 2.5 g of peptone, and 17 g of agar powder were added into a conical flask with a capacity of 1 L, and sterile water was added to 975 ml at a constant volume. Afterwards, 1 ml of 5 mg/ml cholesterol ethanol solution, 1 ml of 1 M CaCl_2_, 1 ml of 1 M MgSO_4_, and 25 ml of 1 M potassium phosphate buffer (pH=6.0) were added. To prevent the eggs from hatching into worms that could crawl into the culture medium, the medium surface was inoculated with 1 ~ 2 ml of membrane blocking solution (MBS), and each dish was inoculated with an egg-bearing female adult. The nematodes that propagated in the medium were washed off, superabsorbent polymer (1.5 g/100 ml) was added to retain moisture, and the dishes were stored in a refrigerator at 4℃.

**Optical and scanning electron microscopy**

Fresh heat-killed nematode adults fixed in triethanolamine formalin (TAF) and subsequently processed with glycerin by Seinhorst's method [[24](#文献24)] were used for microscopy observation. Then, the nematodes were observed by a stereoscope (Leica S8AP0) and a light microscope (Leica DM2500), photos were collected, and the data were measured with a custom hardware configuration.

The scanning electron microscope (SEM) used in this study was a HITACHI S-3400N located at China Agricultural University. The adult nematodes used for scanning electron microscopy were rinsed with phosphate-buffered saline (PBS) and fixed at 4°C for 24 hours with 3% glutaraldehyde solution. They were then postfixed with 2% osmium tetroxide solution for 12 hours at 25°C, dehydrated in a graded ethanol series, critical point dried with liquid CO_2_, mounted on SEM stubs, and coated with gold [[25](#文献25)].

According to observations of nematodes under a light microscope and an SEM, we made a hand-drawn diagram of nematode morphology.

**Molecular analysis**

According to the traditional method used in the LEN laboratory [[26](#文献26)], DNA was extracted from pregnant female adults. Each female adult was placed into an Eppendorf (EP) tube consisting of a 20 μl system, including 17.7 μl of PCR-grade water, 2 μl of 10× PCR buffer with MgCl_2_, 0.2 μl of Tween-20, and 0.1 μl of protease K. After the above steps were completed, the EP tube was placed into a freezer at -80°C and frozen for 10 minutes. Then, the EP tube was quickly put into a water bath at 65°C. After 90 minutes of protease K digestion, the temperature was raised to 95°C through the water bath. After 10 minutes in the water bath, the protease K was inactivated.

Then, the EP tube was quickly placed on ice to cool and centrifuged at 12000 r/min for 2 minutes, after which 1 μl of supernatant was used for PCR. The PCR consisted of a 25 μl system including 12.5 μl of Taq mix, 1 μl of preprimer and 1 μl of postprimer, in addition to 1 μl of the DNA template.

The sequencing results were analyzed by the NCBI database, DNAMAN and MEGA X.

**Results**

***Phasmarhabditis zhejiangensis* sp. nov*.***

([Figs. 1](#fig1)–[3](#fig3))

**Measurements**

See [Table 1](#table1).

**Description**

*Male:* The male has six labial sensilla, each two forming a pair for a total of three pairs, and four cephalic sensilla. The head of the male is flat, and the sensilla of the head and lips are not prominent ([Fig. 3A](#fig3)). The male tail is an open bursa with nine pairs of genital papillae (1+1+1+2+1+3) ([Figs. 1G-H](#fig1), [3J](#fig3)). The spicules are 58 μm (48 μm-68 μm) long ([Figs. 1D](#fig1), [3J](#fig3)), and the gubernaculum is “Y” shaped ([Figs. 1D, G, H](#fig1), [2H](#fig2)). The anterior end of the spicules is slightly enlarged and then tapered towards the end. There are two long and tapering cloacal papillae behind the abdomen and adjacent to the genital papillae ([Fig. 3K](#fig3)). The tail is short and conical, extending to a pointed end, as well as bending towards the venter.

*Female:* The female head also has six labial sensilla (a total of three pairs) and four cephalic sensilla, but compared with the male, the female has a more noticeable head and more obvious labial and cephalic sensilla ([Fig. 3B](#fig3)). The body of the female is also more robust than that of the male. The vulva with a transverse slit is located approximately in the middle of the body, and the vulval lips are protruding and amphidelphic ([Figs. 1C](#fig1), [2E](#fig2), [3G](#fig3)). Pairs of ovaries are symmetrical around the vulva. The female thins from the vulva towards both ends. The end of the female tail suddenly thins into a "small tail" with two short, rod-shaped, conspicuous phasmids appearing in pairs ([Figs. 1F](#fig1), [2F](#fig2), [3H](#fig3)). The posterior anus is slightly swollen and slightly wider and longer than the anal body width ([Figs. 1E](#fig1), [2G](#fig2), [3I](#fig3)).

**Denomination**

The name “***zhejiangensis***” is derived from the Zhejiang region, where the nematode was isolated ("zhejiang-"), and the suffix "-ensis".

**Molecular identification**

The ITS, 18S, and 28S sequencing results for *P. zhejiangensis* were blasted against the NCBI database, and the highest identity was 89.81%, 98.06%, and 95.28%, respectively. We deposited these three sequences in GenBank with accession numbers MK542667, MK937097 and MK937096, respectively.

Evolutionary trees of the ITS ([Fig. 4](#fig4)), 18S ([Fig. 5](#fig5)) and 28S ([Fig. 6](#fig6)) sequences were constructed by the neighbor-joining (NJ) method. The three molecular evolutionary trees showed a close evolutionary relationship between *P. zhejiangensis* and the *Phasmarhabditis* group, with high bootstrap support. Thus, we can generally confirm that this nematode belongs to the genus *Phasmarhabditis*. On the basis of the horizontal lengths of the branches of the molecular evolutionary trees, there were some differences between *P. zhejiangensis* and the closest relatives among the neighboring species; therefore, the studied nematode appears to be a new species of the genus *Phasmarhabditis.*

**Discussion**

Accurate identification of nematode species is fundamental to using nematodes as a biocontrol factor and for further research in nematology. Morphological identification and molecular biological analysis are still very common and important methods for the classification of nematodes. In this paper, the main morphological characteristics of the tested nematodes were observed in depth, including the head, tail, vulva of the female, spicule, gubernaculum and genital papillae of the male, among others. Then, molecular biological analysis of ITS rDNA, 18S rDNA and 28S rDNA sequences was carried out, and the nematode was identified as a new species of the genus *Phasmarhabditis*.

Similar to the families *Steinernematidae* and *Heterorhabditidae* of entomopathogenic nematodes, slug-parasitic nematodes, *Phasmarhabditis* spp., are also an important pathogenic factor of agricultural pests [[27](#文献27)]. The genus *Phasmarhabditis* is now known to include *P. hermaphrodita*, *P. neopapillosa*, *P. papillosa*, *P. phasmarhabditis*, *P. huizhouensis*, *P. tawfiki*, *P. mediterranea* and other species [[28](#文献28)-[30](#文献30)]. The tails of male *P. zhejiangensis* and *P. huizhouensis* have the same genital papillae characteristics. In recent years, species of the genus *Phasmarhabditis* have been continuously reported and improved, which provides a basis for studying the relationships within this genus of nematodes as well as a theoretical basis for nematology and nematode taxonomy.

**Acknowledgments**

The authors thank the anonymous reviewers for their valuable comments. This work was financially supported by the Modern Agricultural Industry Technology System (Project No. CARS-28-18).

**References**

1. Jiří Nermuť, Vladimír Půža, Zdenek Mrácek. Bionomics of the slug-parasitic nematode *Alloionema appendiculatum* and its effect on the invasive pest slug *Arion vulgaris*. BioControl. 2019; 64: 697–707.
2. Wilson M J , Glen D M , George S K , Pearce J D, Wiltshire C W. Biological control of slugs in winter wheat using the rhabditid nematode *Phasmarhabditis hermaphrodita*. Annals of Applied Biology. 2008; 125(2): 377-390.
3. Port C M, Port G R. The biology and behaviour of slugs in relation to crop damage and control. Agricultural Zoology Reuiew. 1986; 1 :253-297.
4. Ester A , Rozen, Molendijk L P G. Field experiments using the rhabditid nematode *Phasmarhabditis hermaphrodita* or salt as control measures against slugs in green asparagus. Crop Protection. 2003; 22(5): 689-695.
5. Glen D M , Wilson M J , Brain P , Stroud G. Feeding Activity and Survival of Slugs, Deroceras reticulatum, Exposed to the Rhabditid Nematode, *Phasmarhabditis hermaphrodita*: A Model of Dose Response. Biological Control. 2000; 17(1):73-81.
6. Nash M A, Thomson L J, Hoffmann A A. Slug control in Australian canola: monitoring, molluscicidal baits and economic thresholds. Pest Management Science. 2007; 63(9): 851-859.
7. Jan Kozłowski, Jaskulska M , Maria Kozłowska. Grazing Behaviour of Slugs (Gastropoda: Arionidae, Agriolimacidae) on the Aboveground and Underground Organs of Potato Plants. Potato Research. 2019; 62: 239–251.
8. Port C M, Port G R. The biology and behaviour of slugs in relation to crop damage and control. Agric Zool Rev. 1986; 1: 255–299.
9. Ester A, Trul R. Slug damage and control of field slug (*Deroceras reticulatum* (Müller)) by carvone in stored potatoes. Potato Res. 2000; 43: 253–261.
10. San-Blas, Ernesto. Progress on entomopathogenic nematology research: A bibliometric study of the last three decades: 1980–2010. Biological Control. 2013; 66(2):102-124.
11. Wilson M J, Glen D M, George S K. The rhabditid nematode *Phasmarhabditis hermaphrodita* as a potential biological control agent for slugs. Biocontrol Science and Technology. 1993; 3: 503-511.
12. Wilson M J, Glen D M, George S K, Hughes L A. Biocontrol of slugs in protected lettuce using the rhabditid nematode *Phasmarhabditis hermaphrodita*. Biocontrol Science and Technology. 1995; 5: 233-242.
13. Iglesias J, Castillejo J, Castro R.. Field test using the nematode *Phasmarhabditis hermaphrodita* for biocontrol of slugs in Spain. Biocontrol Science and Technology. 2001a; 11: 93-98.
14. Iglesias J, Castillejo J, Castro R. Mini-plot field experiments on slug control using biological and chemical control agents. Annals of Applied Biology. 2001b; 139: 285-292.
15. Vernavá M N, Phillips A P, M, Hughes L, RowcliffeH, WiltshireC, Glen D. Influences of preceding cover crops on slug damage and biological control using *Phasmarhabditis hermaphrodita*. Annals of Applied Biology. 2005; 145: 279-284.
16. Tandingan De Ley I, McDonnell RD, Lopez S, Paine TD, De Ley P. *Phasmarhabditis hermaphrodita* (Nematoda: Rhabditidae), a potential biocontrol agent isolated for the first time from invasive slugs in North America. Nematology. 2014; 16: 1129–1138.
17. Hooper DJ, Wilson MJ, Rowe JA, Glen DM. Some observations on the morphology and protein profiles of the slug-parasitic nematodes *Phasmarhabditis hermaphrodita* and *P. neopapillosa* (Nematoda: Rhabditidae). Nematology. 1999; 1: 173–182.
18. Mengert H. Nematoden und Schnecken. Zeitschrift für Morphologie und Ökologie der Tiere. 1953; 4: 311–349.
19. Poinar GO, Jr. Origins and phylogenetic relationships of the entomophilic rhabditids, *Heterorhabditis* and *Steinernema*. Fundam appl Nematol. 1993; 16: 333–338.
20. Azzam K M. Description of the nematode *Phasmarhabditis tawfiki* n. sp. isolated from Egyptian terrestrial snails and slugs. J Egypt Ger Soc Zool. 2003; 42: 79–87.
21. Jiří Nermuť, Vladimír Půža, Mekete T , Mráček Z. *Phasmarhabditis bonaquaense* n. sp. (Nematoda: Rhabditidae), a new slug-parasitic nematode from the Czech Republic. Zootaxa. 2016; 4179(3): 530-546.
22. Mc Donnell R J , De Ley P , Irma T D L , Holovachov O, Paine T D, Win B. Description of *Phasmarhabditis californica* n. sp. and first report of *P. papillosa* (Nematoda: Rhabditidae) from invasive slugs in the USA. Nematology. 2016; 18(2): 175-193.
23. Brenner S. 1974. The genetics of *Caenorhabditis elegans*. Genetics, 77, 71-94.
24. Seinhorst, J. W . A Rapid Method for the Transfer of Nematodes From Fixative To Anhydrous Glycerin[J]. Nematologica, 1959, 4(1):67-69.
25. Nguyen K B , Smart G C . Morphometrics of Infective Juveniles of *Steinernema* spp. and *Heterorhabditis bacteriophora* (Nemata: Rhabditida). Journal of Nematology, 1995, 27(2):206-12.
26. Xingyue L , Qizhi L , Vladimír Půža, JIIÍ NERMUŤ, ZDENĚK MRÁČEK. *Heterorhabditis beicherriana*, n. sp. (Nematoda: Heterorhabditidae), a new entomopathogenic nematode from the Shunyi district of Beijing, China. Zootaxa, 2012, 3569(3569):25-40.
27. Grewal P S, Ehlers R U, Shapiro-Ilan D I. Nematodes as biocontrol agents. 2005.
28. Tandingan De Ley I, McDonnell RD, Lopez S, Paine TD, De Ley P. *Phasmarhabditis hermaphrodita* (Nematoda: Rhabditidae), a potential biocontrol agent isolated for the first time from invasive slugs in North America. Nematology. 2014; 16: 1129-1138.
29. Rowe J , Wilson M , Glen D , Hooper D. Some observations on the morphology and protein profiles of the slug-parasitic nematodes *Phasmarhabditis hermaphrodita* and *P. neopapillosa* (Nematoda: Rhabditidae). Nematology. 1999; 1(2):173-182.
30. Huang R E , Ye W , Ren X , Zhao Z. Morphological and Molecular Characterization of *Phasmarhabditis huizhouensis* sp. nov. (Nematoda: Rhabditidae), a New Rhabditid Nematode from South China. Plos one. 2015; 10(12).

**Fig. 1 Hand drawings of *Phasmarhabditis zhejiangensis* sp. nov.** (A) Head of female, (B) Head of male, (C) Vulva of female, (D) Spicule and gubernaculum, (E) Lateral view of female tail, (F) Ventral view of female tail, (G) Lateral view of male tail, (H) Ventral view of male tail.

**Fig. 2 Optical micrographs** **of *Phasmarhabditis zhejiangensis* sp. nov.** (A) Male, (B) Female, (C) Head of female, (D) Head of male, (E) Vulva, (F) Lateral view of female tail, (G) Ventral view of female tail, (H) Spicule and gubernaculum, (I) Spicule, (J and K) Ventral view of male tail, (L) Lateral view of male tail.

**Fig. 3 Scanning electron micrograph of *Phasmarhabditis zhejiangensis* sp. nov.** (A) Head of male (LS: labial sensilla; CS: cephalic sensilla), (B) Head of female, (C) Enlarged view of excretory pore, (D) Excretory pore in the anterior region of the body in both the male and female, (E) Lateral field of female, (F) Lateral field of male, (G) Lateral view of the vulva of the female, (H) Positive view of the female tail (ph: phasmid), (I) Lateral view of the female tail, (J) Lateral view of the male tail, (K) Ventral view of the male tail (cp: cloacal papillae).

**Fig. 4 Molecular evolutionary tree based on the ITS sequence of *Phasmarhabditis zhejiangensis* sp. nov.**

**Fig. 5 Molecular evolutionary tree based on the 18S rDNA sequence of *Phasmarhabditis zhejiangensis* sp. nov.**

**Fig. 6 Molecular evolutionary tree based on the 28S rDNA sequence of *Phasmarhabditis zhejiangensis* sp. nov.**

**Table 1** **Morphological data of *Phasmarhabditis zhejiangensis sp. nov.*** All measurements are presented in μm and in the form mean ± s. d. (range) (n=20).

| Character | Females | Males |
| --- | --- | --- |
|  | (n=20) | (n=20) |
| L | 1911.420±294.544  (1440.690-2354.877) | 1486.351±241.718  (1014.584-1842.650) |
| a | 16.611±1.209  (14.092-18.790) | 20.272±2.009  (17.002-25.647) |
| b | 7.717±0.850  (6.253-10.067) | 7.497±0.882  (5.633-8.836) |
| c | 26.025±4.720  (19.869-33.521) | 31.028±7.003  (20.012-48.671) |
| c’ | 1.308±0.261  (0.933-1.885) | 1.148±0.182  (0.838-1.429) |
| V | 53.943±1.367  (50.838-57.193) | — |
| Greatest body diameter | 115.070±19.548  (79.130-154.686) | 74.257±15.044  (50.917-94.098) |
| Esophageal length | 247.691±21.483  (214.210-285.517) | 197.324±12.258  (176.277-223.838) |
| Distance from anterior end of body to nerve ring | 195.038±18.746  (166.880-228.531) | 161.677±14.111  (133.940-179.065) |
| Distance from anterior end of body to excretory pore | 243.672±22.858  (201.650-289.525) | 208.799±19.183  (179.396-237.649) |
| Distance from anterior end of body to vulva | 1031.080±152.766  (783.670-1264.181) | — |
| Distance from vulva to anus | 809.886±145.286  (549.251-1013.156) | — |
| Anal body diameter | 56.132±10.182  (35.784-75.278) | 43.502±8.348  (30.670-62.027) |
| Tail length | 73.447±6.295  (59.850-83.486) | 49.881±12.708  (31.248-88.629) |
| Length of female pointed tip | 35.573±5.003  (24.766-47.445) | — |
| Spicule length | — | 58.005±5.100  (48.268-68.108) |
| Gubernaculum length | — | 20.117±2.643  (15.697-27.982) |
| Gubernaculum length (% of spicule length) | — | 34.775±4.039  (25.577-42.834) |
