# Supplementary material for: Phasmarhabditis zhejiangensis sp. nov. (Nematoda: Rhabditidae), a new rhabditid nematode from Zhejiang, China
Source: PLoS One. 2020 Nov 9;15(11):e0241413. doi: 10.1371/journal.pone.0241413 (PMC7652337; doi:10.1371/journal.pone.0241413)
Supplement: S1 File — (ZIP) [file pone.0241413.s001.zip › supporting information/Z3H93XY7_coverletter_edited_final.docx]

Dear Editor,

Attached is our paper, which we are submitting to PLoS ONE.

Title: *Phasmarhabditis zhejiangensis* sp. nov. (Nematoda: Rhabditidae), a new rhabditid nematode from Zhejiang, China

Authors: Chao-nan ZHANG, Qi-zhi LIU^*^

Nematodes are commonly used as effective biological protection factors. However, nematodes that can be used to kill slugs have rarely been found. Therefore, the identification of new nematode species that are harmful to slugs is significant. In this paper, we isolated a nematode, *Phasmarhabditis zhejiangensis* sp. nov., from the surface of a slug (*Philomycus bilineatus* Bonson). *P. zhejiangensis* was isolated and identified by morphological analysis and molecular analysis, which provides an important basis for biological control of slugs and biodiversity research.

None of the material in this paper has been published or is under consideration for publication elsewhere.

The corresponding author is Prof. Liu Qizhi, and her address and other information are as follows:

Address: College of Plant Protection, China Agricultural University, 2 Yuanmingyuan West Road, Haidian District, Beijing, China.

E-mail: lqzzyx163@163.com

Tel: 86-18519264197

We would greatly appreciate if you could guide us in editing our manuscript to meet your journal’s quality standards.

Yours sincerely,

Prof. Liu Qizhi
